# Rumen fluke (*Calicophoron daubneyi*) on Welsh farms: prevalence, risk factors and observations on co-infection with *Fasciola hepatica*

**DOI:** 10.1017/S0031182016001797

**Published:** 2016-11-01

**Authors:** RHYS ALED JONES, PETER M. BROPHY, E. SIAN MITCHELL, HEFIN WYN WILLIAMS

**Affiliations:** 1 Institute of Biological, Environmental and Rural Sciences (IBERS), Aberystwyth University, Penglais, Abersystwyth, Ceredigion, UK; 2 Animal and Plant Health Agency (APHA), Carmarthen Veterinary Investigation Centre, Job's Well Rd, Johnstown, Carmarthen SA31 3EZ, UK

**Keywords:** *Calicophoron daubneyi*, *Fasciola hepatica*, co-infection, cattle, sheep, logistic regression model, null modelling, UK

## Abstract

Reports of *Calicophoron daubneyi* infecting livestock in Europe have increased substantially over the past decade; however, there has not been an estimate of its farm level prevalence and associated risk factors in the UK. Here, the prevalence of *C. daubneyi* across 100 participating Welsh farms was recorded, with climate, environmental and management factors attained for each farm and used to create logistic regression models explaining its prevalence. Sixty-one per cent of farms studied were positive for *C. daubneyi*, with herd-level prevalence for cattle (59%) significantly higher compared with flock-level prevalence for sheep (42%, *P* = 0·029). Co-infection between *C. daubneyi* and *Fasciola hepatica* was observed on 46% of farms; however, a significant negative correlation was recorded in the intensity of infection between each parasite within cattle herds (rho = −0·358, *P* = 0·007). Final models showed sunshine hours, herd size, treatment regularity against *F. hepatica*, the presence of streams and bog habitats, and Ollerenshaw index values as significant positive predictors for *C. daubneyi* (*P* < 0·05). The results raise intriguing questions regarding *C. daubneyi* epidemiology, potential competition with *F. hepatica* and the role of climate change in *C. daubneyi* establishment and its future within the UK.

## INTRODUCTION

Rumen fluke (Paramphistomatidae spp.) are trematode parasites infecting ruminants worldwide. Traditionally rumen flukes were regarded as parasites mainly confined to tropical and sub-tropical areas. However, within European livestock, the presence in recent decades of high levels of rumen fluke, in particularly the species *Calicophoron daubneyi,* is of potential concern. *Calicophoron daubneyi* was first recorded infecting cattle in Kenya in the 1950s (Dinnik, 1962), with confirmation of its occurrence in Europe from the 1970s (Sey, 1980) and the UK in 2012 (Gordon *et al.* 2012). In recent years, the UK has experienced an apparent sudden increase in the prevalence of rumen fluke, with the proportion of rumen fluke detected in ruminant submissions by passive veterinary surveillance increasing on average by 57% annually between 2010 and 2015 (VIDA, 2016*a*). High prevalence has been observed across Western Europe, with abattoir studies recording cattle prevalence levels of 29% in the UK (Sargison *et al.* 2016), 44·7% in France (Mage *et al.* 2002), 18·8% in Spain (Gonzalez-Warleta *et al.* 2013), 28% in Belgium (Malrait *et al.* 2015) and 52% in Ireland (Toolan *et al.* 2015). Paramphistomosis (rumen fluke disease) has been reported in both cattle (Millar *et al.* 2012) and sheep (Mason *et al.* 2012) in the UK, however, UK passive veterinary surveillance has been detecting rumen fluke in larger proportions of cattle submissions compared with sheep submissions (VIDA, 2016*a*), while in Ireland prevalence levels have been shown to be lower in sheep compared with cattle (Toolan *et al.* 2015). In all cases of paramphistomosis, a heavy burden of juvenile fluke in the intestine has been attributed as the cause of the disease, with adult *C. daubneyi* believed to be well tolerated (Zintl *et al.* 2014). Nevertheless, adult *C. daubneyi* are known to induce inflammatory reactions in the rumen and reticulum (Fuertes *et al.* 2015), and in some instances may cause symptoms including bloat, loss of condition and the softening of feces in infected cattle (Alzieu and Dorchies, 2007). The potential threat of these symptoms to the UK livestock industry is heightened due to limited anthelmintic options for treatment, with oxyclozanide the only anthelmintic regarded as an effective paramphistomicide (Malrait *et al.* 2015). All published molecular level studies highlight *C. daubneyi* as the dominant and potentially the only Paramphistomatidae species present in UK livestock (Gordon *et al.* 2013; Huson *et al.* 2015), while earlier reports of *Paramphistomum cervi* in British livestock were only based on morphological identification (Pillers, 1922; Craig and Davies, 1937). Recent molecular analyses have also identified *Paramphistomum leydeni* infecting reindeer in south west England (VIDA, 2016*b*) and fallow deer in Ireland (O'Toole *et al.* 2014), leading to the possibility of alternative Paramphistomatidae spp. infecting UK livestock.

As with other trematode species, rumen fluke requires an intermediate snail host to complete its lifecycle, a process which sees the parasite exploit this host to develop and multiply rapidly. The prominent host of *C. daubneyi* is *Galba truncatula* (Dinnik, 1962; Degueurce *et al.* 1999; Martinez-Ibeas *et al.* 2013), a snail species, which thrives in the UKs consistently wet and mild climate. *Galba truncatula* is also the prominent intermediate host of the highly pathogenic liver fluke (*Fasciola hepatica)*, a parasite which has been endemic in the UK for centuries (Dalton, 1998). As *G. truncatula* has recently been shown to host *C. daubneyi* in the UK (Jones *et al.* 2015), the potential epidemiological range of this parasite is also likely to be widespread.

Numerous predictive models of *F. hepatica* based on key climatic drivers for *G. truncatula* activity, including rainfall and temperature have been created over the past 60 years. The most prominent model, the Ollerenshaw index (Ollerenshaw and Rowlands, 1959), is widely used commercially as a regional fasciolosis risk guide for farmers (NADIS, 2016). In recent years, further climatic *F. hepatica* models have been created with the inclusion of either or both environmental factors (McCann *et al.* 2010) and farm management factors (Howell *et al.* 2015) in an attempt to predict fasciolosis occurrence at a finer scale. As well as increasing the accuracy of models, any farm management practices identified as risk factors for *F. hepatica* may inform veterinarians and farmers of best practices to negate fasciolosis risk. However, despite their similar reliance on *G. truncatula,* it remains to be resolved whether these models can accurately predict the occurrence of *C. daubneyi* due to differences in their epidemiology including, different egg hatching stimulus (Chryssafidis *et al.* 2015), timeframe of development within the snail (Dreyfuss, 2015), sensitivity to temperature at shedding (Abrous *et al.* 1999) and geotropism of cercariae (Dreyfuss *et al.* 2004). These epidemiological differences mean risk factors for *C. daubneyi* presence may vary significantly from *F. hepatica,* although risk factors for *G. truncatula* occurrence are likely to be confounding risk factors for both parasites. *Calicophoron daubneyi* models are currently scarce compared with models for *F. hepatica*. A Galician model identified decreasing rainfall and temperature, and increasing cattle density and slope as predictors of *C. daubneyi* prevalence in cattle (Gonzalez-Warleta *et al.* 2013), while an Italian Apennines model identified the presence of streams, springs or brooks, heathland and moorland as positive predictors of *C. daubneyi* (Cringoli *et al.* 2004). In these countries, the climate, environment and agricultural systems are very different to the UK, and risk factors associated with *C. daubneyi* prevalence is therefore likely to vary within each country. There are also unanswered questions regarding the potential interaction between *C. daubneyi* and *F. hepatica* at intermediate host level in the UK (Jones *et al.* 2015), which could influence each parasite's distribution in the presence of the other. This consequence could be positive, due to a synergistic effect in infecting alternative snail species (Abrous *et al.* 1998), or negative, due to within-snail predation and competition for nutrients (Rondelaud *et al.* 2007).

In this case study, the prevalence of *C. daubneyi* on participating Welsh farms was recorded along with climatic, environmental and farm management factors for each farm. The aim was to create models explaining the presence of *C. daubneyi* on Welsh farms and to identify associated risk factors. *Fasciola hepatica* prevalence was also recorded with the aim of comparing the prevalence, infection intensity and risk factors of both parasites.

## MATERIALS AND METHODS

### Questionnaire

During 2015 farmers were invited to participate in the study through Young Farmers Clubs Wales, on social media and at various agricultural events. From September 2015 participants were instructed to fill an online questionnaire (Schmitz, 2015) containing 36 questions regarding the number and type of livestock on their farm, the presence of liver fluke and rumen fluke on their farm, their farms environmental features and management factors. Twenty-nine questions were closed, and seven were open ended. Open ended questions included months when cattle were housed, months of liver fluke and rumen fluke treatment, and which anthelmintics were used to treat against each parasite. In the case of anthelmintic use, answers given as the product brand name were transformed into the active drug class for that particular product. Participants were also asked to provide their full postal address and the postcode district location of each of their major holdings along with their proportional size. In total of 128 farmers completed the survey, all of which indicated their willingness to test their livestock for fluke. The project was approved by the Aberystwyth University Research Ethics Panel (project number 496).

### Fecal sampling and testing

A total of 128 packages containing gloves, 25 mL measuring apparatus, 50 mL tubes, question sheet and a prepaid return envelope were dispatched, and 100 were returned with appropriate samples between November 2015 and February 2016. Participants were instructed to collect 25 mL of feces from 20 individual cattle or sheep using a supplied measuring apparatus, before thoroughly mixing the samples and using the same apparatus to place 25 mL of feces in a supplied tube for posting to the laboratory. Farmers with both cattle and sheep were requested to repeat the process for both species and return separate 25 mL samples. The amount of feces requested for submission was selected to ensure return packages conformed to UN3373 biological sample postage regulations. Participants were also invited to submit details of sampled animals including, their age and most recent anthelmintic treatment against fluke species. Samples were stored at 4 °C and processed within 48 h of arrival at the laboratory. Fecal samples were tested for fluke infection using a sedimentation fecal egg count (FEC) technique. Approximately 20 g of feces were mixed thoroughly with water and washed through 300, 150 and 45 *µ*m mesh sieves. Materials including any fluke eggs collected on the 45 *µ*m sieves were washed into a 1 L measuring cylinders and allowed to sediment for 7 min. The supernatant was removed via aspiration, with the process repeated 3–4 times until the sample was sufficiently clear. The samples were then stained with two drops of 1% methylene blue, placed in a 5 cm petri dish and viewed under a stereo microscope to count fluke eggs. Fluke eggs were differentiated via the golden and clear colour of *F. hepatica* and *C. daubneyi* eggs, respectively (Kajugu *et al.* 2015), before the numbers of eggs counted were divided with the weight of feces tested to calculate the eggs per gram (EPG) values.

### Identification of C. daubneyi in rumen fluke positive cases

DNA from rumen fluke eggs that were isolated from feces via FEC were used to confirm the presence of *C. daubneyi* in each positive sample. Eggs and debris were resedimented until concentrated into a 0·5 mL centrifuge tube. If very low levels of eggs were observed during the FEC, the eggs were pipetted from the final FEC sedimentation to a 0·5 mL centrifuge tube. Four 0·1–0·5 mm zirconia beads and 100 *µ*L of 5% Chelex^®^ 100 (Bio-Rad, Hercules, USA) was added to the mixture and vortexed for 2 min, prior to incubation firstly at 56 °C for 60 min, and secondly at 95 °C for 10 min. Samples were centrifuged at 15 000 rpm for 7 min with the supernatant diluted to the required concentration with nuclease free water (1/10 or 1/100). Each DNA extraction sample was subjected to an in-house polymerase chain reaction (PCR) protocol using *C. daubneyi* specific primers (F:5′-GTTTGTGTGGTTTGCCACGG-3′; R:5′-CTACCCCAAGCAGCCACTAC-3′) that amplifies 169 bp strands from the *C. daubneyi* cytochrome *c* oxidase subunit 1 (*cox*1) gene (GenBank JQ815200). Primers were designed using Geneious software (Kearse *et al.* 2012) and were based on regions of the *cox1* gene unique to *C. daubneyi* in comparison with other Paramphistomatidae spp. and *F. hepatica.* Designed primer sequences were cross-referenced with NCBI sequences via primer-BLAST to ensure species specificity for its amplified sequences. For each sample, a 25 *µ*L master mix was created containing 12·5 *µ*L of MyTaq^™^ red mix (Bioline, London, UK), 50 *µ*m of primer, 1 *µ*L of the extracted DNA and nuclease free water. Each sample was subjected to PCR amplification consisting of an initial denaturation at 95 °C for 2 min followed by 35 cycles consisting of stages of denaturation (30 s at 95 °C) annealing (30 s at 65 °C) and extension (30 s at 72 °C), before a final 10 min extension phase at 72 °C. PCR products were visualized in 1% agarose gel stained with GelRed (Biotium, Hayward, USA) along with positive and negative controls, with 169 bp band visualized in a well under UV light signifying a positive species identification for *C. daubneyi*. The presence or absence of other Paramphistomatidae spp. in sampled animals was not confirmed.

### Rumen fluke and liver fluke prevalence

All statistical tests described in this section were performed in SPSS (v. 22). Prevalence data at farm, flock and herd level were attained by calculating the proportion of samples with EPG levels greater than zero at each respective level. To calculate the prevalence of *C. daubneyi* in participating farms from different regional areas, farms were categorized into six regions (north west, north east, Ceredigion, Montgomery, south west, south east) created using ordnance survey boundary line data (Ordnance Survey, 2016) in ArcMap (v 10.2.2). A chi-square test was performed to compare *C. daubneyi* prevalence levels on farms in western regions (north west, Ceredigion, south west) and eastern regions (north east, Montgomery, south east) of Wales. Differences in the prevalence of both *C. daubneyi* and *F. hepatica* between cattle herds and sheep flocks were analysed using a chi-square test. A Mann–Whitney U-test used to analyse differences in EPG levels of both parasites between cattle herds and sheep flocks for all cases where EPG levels were >0. As numerous farms in this study only had one livestock species present on their holding, the results of the two latter analyses could be skewed due to potential differences in the type of land seen on sheep only farms compared with cattle only farms. To combat this issue, parasite prevalence and intensity data from farms submitting samples for both livestock species were also analysed using paired difference tests (referred to as ‘paired’ samples hereafter). This allowed the prevalence and infection intensity of each parasite to be compared between cattle and sheep on each individual farm. This was analysed using a McNemar test (McNemar, 1947) for positive/negative cases, and a Wilcoxon ranked signed test for EPG values. The intensity of *F. hepatica* and *C. daubneyi* infections within herds, flocks and farms was also compared using spearman rank correlation (rho). Only herds and flocks, which were positive for either or both parasites were included in the analyses.

### Data sources

Variables regarding farm structure, management and observed environmental features were extracted from questionnaire answers in Microsoft Excel. In instances where questions were directed at a specific species (cattle or sheep), answers were also adapted to be representative of farm level. For example, on farms where both cattle and sheep were present, the mean number of yearly *F. hepatica* treatments was calculated. Further variables were calculated using data from the questionnaire, including cattle grazing season length, grazing density (LSU/ha) and timing of *F. hepatica* treatment. Questionnaire postcode data were also used to geo-reference participating farms using ArcGIS (v 10.2.2). A full postcode for each farms address, or their largest holdings where applicable, was derived from the questionnaire and converted into geographical coordinates.

Observed climate data were sourced from the Met Office at 5 km^2^ resolution (Perry and Hollis, 2005). A detailed literature review was performed to identify potential climate factors on the *C. daubneyi* lifecycle, with each identified factor calculated from monthly observed data. For each variable, a value for the year 2015 and a value sourced from 2011 to 2015 (2012–2015 in the instance of sunshine hours due to changes in data format) were calculated. Climatic data were also used alongside extra-terrestrial radiation data (Duffie and Beckman, 2013) to calculate 5 km^2^ resolution Ollerenshaw index values (Mt) (Ollerenshaw and Rowlands, 1959) during the last 12 months and between 2011 and 2015.

Data regarding soil type, pH and moisture levels at 1 km^2^ resolution were sourced from the Centre for Ecology and Hydrology (CEH) (Henrys *et al.* 2012; Henrys *et al.* 2014), and soil mineral levels were sourced from the British geological survey (BGS) (Rawlins *et al*. 2012). A literature review of minerals shown to either have been included in predictive models for *F. hepatica* or known to have an effect on Lymnaeidae snail biology determined the choice of soil minerals to analyse. ArcMap (v 10.2.2) was used to extract all climate and environmental data using the raster to point function.

### Statistical analysis of *C. daubneyi* and *F. hepatica* presence

Presence data for *C. daubneyi* and *F. hepatica* along with the data sourced above was statistically analysed with the aim of creating models to explain the presence and absence of the parasites on the study farms, cattle herds and sheep flocks.

#### Univariate analysis

Variables regarded as being potential predictor values for *C. daubneyi* and *F. hepatica* were selected for inclusion as potential model variables via univariate analysis. Univariate analysis was performed using the Pearson chi-square test for binary variables and the Mann–Whitney U-test for numerical variables in SPSS (v 22·0). For each test, a significance value of *P* < 0·10 was required for each variable to be selected for the next stage of analysis.

#### Logistic regression

Binary logistic regression was performed on the data from the univariate analysis to create candidate models explaining the presence or absence of *C. daubneyi*. Farms with *C. daubneyi* EPG values >0 for either or both cattle and sheep samples were considered as *C. daubneyi* positive, while farms with *C. daubneyi* EPG of 0 from all samples were considered negative. Forward Wald and backward Wald logistic regression with a probability for stepwise of *P* < 0·05 for entry and *P* > 0·10 for removal was performed in SPSS (v.22) to create candidate models. Odds ratios and their respective 95% confidence intervals were calculated to quantify the strength of each variable's association with *C. daubneyi* prevalence within each model. However, when prevalence is above 10%, odds ratios can overestimate risk (Zhang and Yu, 1998) and thus any interpretation of odds ratios in these instances must be made cautiously. This process was repeated to create cattle herd (*n* = 76) and sheep flock (*n* = 90) *C. daubneyi* models. The above process was also repeated to create a farm level *F. hepatica* model. However, as 14 farms had recently treated all sampled animals with flukicide prior to submission of their FEC samples, only 86 farms were used to create this model.

#### Multi-model selection and fitting

Each candidate model was tested for goodness of fit via the Hosmer–Lemeshow test with non-significant chi-square statistic values (*P* > 0·05) indicating a model with goodness of fit. The small sample corrected Akaike information criterion (AICc) (Burnham and Anderson, 2002) was calculated with the SAM (v4.0) (Rangel *et al.* 2010) software for candidate models identified by stepwise model building. The model with the lowest AICc values were considered the models with the most empirical support (Burnham and Anderson, 2002) and chosen for further predictive testing.

#### Model predictive testing

Interpreting the predictive performance of logistic regression models through metrics such as the area under the curve (AUC) is an important part of their evaluation (Fielding and Bell, 1997), but one fraught with dangers such as the inflation of metrics by spatial patterns in the data leading to inflated Type I errors (Lobo *et al.* 2008). Consequently, the AUC values of the chosen models were compared against appropriate null models using the method proposed by Beale *et al*. (2008) and adapted by Williams *et al*. (2015). For each selected model, 99 null models were created and these null models retained the spatial patterns in the observed distribution of *C. daubneyi* on farms. In each instance, models were randomly split 1000 times into training and testing points at a ratio of 60:40, respectively. The median AUC of each test model across 1000 splits were then calculated. The chosen models were adjudged to have identified a prediction greater than expected by chance if their median AUC were higher than the median AUC values of 95 of accompanying null models. See Williams *et al*. (2015) for full details of the methodology used.

## RESULTS

### Descriptive statistics

One hundred farms submitted samples for testing the presence or absence of *C. daubneyi*. Details on the number of herd types and flocks along with the average size of each can be seen in Table 1.

**Table 1.** Descriptive statistics regarding the number of participating farms, herds and flocks and their mean size

	*n* farms	Mean size/animal *n*	Range
Farm size			
Farm area (ha)	100	138	3–480
Farm enterprises			
Dairy herds (adult cattle)	8	204	60–550
Suckler cow herds (adult cattle)	62	36	5–150
Heifer/steers herds (>12 months of age)	70	68	5–1100
Sheep flocks (adult sheep)	85	595	50–4000

Forty-nine per cent of participants were aware of rumen fluke prior to this study, with 10% indicating rumen fluke was or had been present on their farms. There was no significant relationship between a participants prior knowledge of rumen fluke and its presence on their farm (χ^2^ = 0·749, *P* = 0·387). Six per cent of participating farmers had recently treated directly against rumen fluke with an oxyclozanide product, with 5% using the anthelmintic to treat against *F. hepatica* over the past 12 months.

### Rumen fluke species ID

DNA from 81 rumen fluke egg samples (44 cattle; 37 sheep) were extracted and screened for *C. daubneyi* DNA. All 81 samples (100%) were positive for *C. daubneyi.*

### Prevalence

**Fig. 1.**
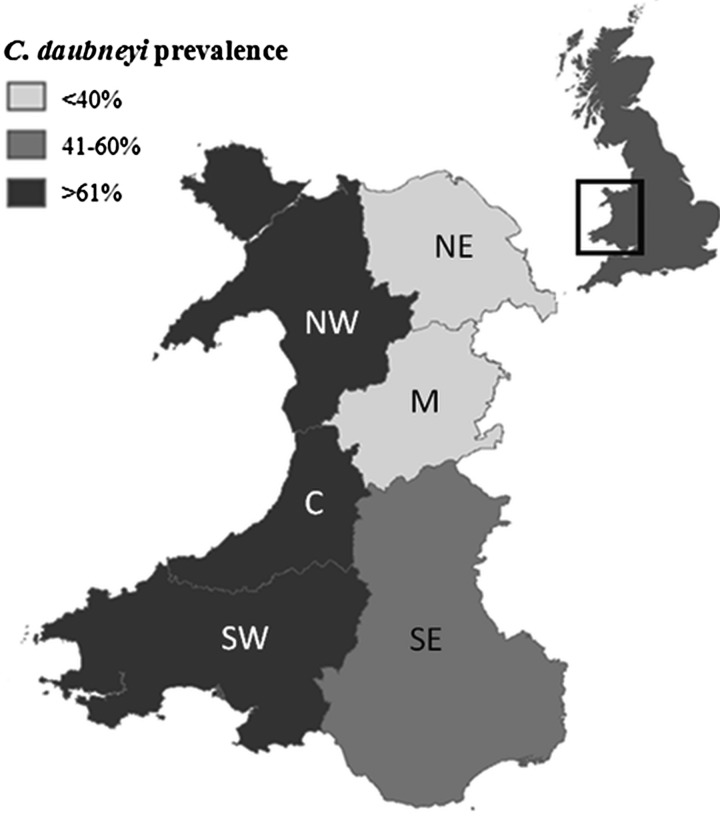
Prevalence of *C. daubneyi* in regional areas of Wales: NW – north west (*n* = 19) NE – north east (*n* = 14), C – Ceredigion (*n* = 19), M – Montgomery (*n* = 13), SW – south west (*n* = 15), SE – south east (*n* = 20). Prevalence of *C. daubneyi* was significantly higher (χ^2^ = 7·507, *P* = 0·006) in western regions (NW, C, SW) (73·6%) compared with eastern regions (NE, M, SE) (46·8%). Contains OS data^©^ Crown copyright and database right (2016).

Sixty-one per cent of farms were positive for *C. daubneyi,* while 68% were positive for *F. hepatica,* with only 17% of farms negative for both. Co-infection of both *C. daubneyi* and *F. hepatica* was seen on 46% of farms. The prevalence of *C. daubneyi* across six regional areas of Wales can be seen in Fig. 1. *Calicophoron daubneyi* prevalence was significantly higher in western regions (NW, C, SW) of Wales compared with eastern regions (NE, M, SE) (χ^2^ = 7·507. *P* = 0·006).

The prevalence of *C. daubneyi* and *F. hepatica* in herds and flocks are presented in Table 2. Fifty-nine per cent of cattle herds sampled in this study were positive for *C. daubneyi,* compared with a significantly lower prevalence level of 42% in sampled sheep flocks (*P* = 0·029). The prevalence levels of *C. daubneyi* in paired cattle herds (59%) was significantly higher compared with their sheep flock counterparts (42%) when using a paired difference test (*P* = 0·035) on individual farms, which had submitted both cattle and sheep samples. There was no significant difference between the EPG levels of *C. daubneyi* within positive sheep flocks and cattle herds in either normal (*P* = 0·596) or paired (*P* = 0·131) analysis (Table 3).

**Table 2.** Prevalence of *C. daubneyi* and *F. hepatica* within cattle herds and sheep flocks in both the total submitted samples and paired samples

	Total samples			Paired samples		
	Prevalence (*n*)	χ^2^	Sig.	Prevalence (*n*)	χ^2^	Sig.
*Fasciola hepatica* cattle	55% (76)	0·11	0·916	58% (66)	0·36	0·851
*Fasciola hepatica* sheep	54% (90)			55% (66)		
*Calicophoron daubneyi* cattle	59% (76)	4·76	0·029	59% (66)	7·63	0·035
*Calicophoron daubneyi* sheep	42% (90)			42% (66)

**Table 3.** Mean EPG levels for *C. daubneyi* and *F. hepatica* in positive cattle herds and sheep flocks in both the total submitted samples and paired samples

		Total samples				Paired samples			
	Max EPG	Mean EPG (*n*)	s.d.	U	Sig.	Mean EPG (*n*)	s.d.	*Z*	Sig.
*Fasciola hepatica* EPG cattle	5	0·96 (42)	1·1	399	0·000	1·02 (23)	1·19	−2·92	0·004
*Fasciola hepatica* EPG sheep	300	19·76 (49)	49·32			22·89 (23)	32·49		
*Calicophoron daubneyi* EPG cattle	70	9·94 (45)	14·39	−530	0·596	11·37 (22)	12·93	−1·51	0·131
*Calicophoron daubneyi* EPG sheep	113	9·93 (38)	20·44			8·99 (22)	12·62		

EPG, eggs per gram.

There was no significant difference between prevalence levels of *F. hepatica* in cattle herds and sheep flocks in the total submitted samples (*P* = 0·916) and paired (*P* = 0·851) samples (Table 2). *Fasciola hepatica* EPG level were however, significantly higher in positive sheep flocks compared with positive cattle herds for total samples (*P* < 0·001) and paired samples (*P* = 0·004) (Table 3).

### Fluke species correlation

A significant negative correlation between EPG levels of *C. daubneyi* and *F. hepatica* was recorded for herds (rho = −0·358, *P* = 0·007), with a non-significant (rho = −0·199, *P* = 0·11) negative correlation recorded for flocks.

### Logistic regression models of *C. daubneyi* and *F. hepatica* presence or absence

Univariate analysis selected 20, 24 and 14 variables as potential predictors of *C. daubneyi* at farm, herd and flock level, respectively, for input into logistic regression analysis (Supplementary Table 1). The final models explaining the prevalence of *C. daubneyi* on Welsh farms, in cattle herds and sheep flocks are seen in Table 4. The farm model identified four positive predictors for *C. daubneyi* on Welsh farms; the cattle model identified five positive predictors for *C. daubneyi* in cattle herds, while the sheep model identified two positive predictors for *C. daubneyi* in sheep flocks. Univariate analysis selected 17 variables as potential predictors of *F. hepatica* at farm level, and four significant predictors were selected in the final model (Table 4). All final models AICc values were at least two points lower than the AICc values of other candidate models, including models created using only climate variables.

**Table 4.** Logistic regression models explaining the prevalence of *C. daubneyi* on Welsh farms, and in cattle herds, and sheep flocks

Model	Variable	HL	AUC	AICc	NMR	B	s.e.	Wald	Sig.	Odds Ratio	Lower 95% CI	Upper 95% CI
Farm *C. daubneyi*		0·78	0·85	103·2	1st							
	Constant					−15·022	3·663	16·87	0	0	–	–
	Number of heifers/steers (Over 12 months)					0·03	0·009	10·66	0·001	1·03	1·012	1·049
	Mean annual treatment of livestock against *F. hepatica*					1·359	0·479	8·06	0·005	3·89	1·523	9·941
	Presence of streams or drainage ditches					3·997	1·453	7·57	0·006	54·46	3·154	940·12
	Presence of bog habitats					1·342	0·735	3·34	0·068	3·83	0·906	16·169
	Mean daily sunshine hours: MJJ (2015)					1·308	0·479	8·08	0·004	3·7	1·501	9·12
Cattle *C. daubneyi*		0·74	0·86	86·2	1st							
	Constant					−17·867	4·922	13·18	0	0	–	–
	Number of cattle over 12 months					0·009	0·004	3·67	0·055	1·009	1	1·017
	Treatment of cattle against *F. hepatica* in spring					2·808	1·19	5·56	0·018	16·57	1·607	170·85
	Presence of bog habitats					1·714	0·77	4·96	0·026	5·55	1·228	25·108
	Water flowing from other farm					2·019	0·986	4·19	0·041	7·53	1·091	51·99
	Mt Summer 2015					0·013	0·005	6·52	0·011	1·013	1·003	1·024
	Sunshine hours: MJJ (2012–2015)					1·565	0·654	5·73	0·017	4·79	1·328	17·226
Sheep *C. daubneyi*		0·52	0·80	99·3	1st							
	Constant					−14·279	3·977	12·89	0	0	–	–
	Number of heifers/steers (over 12 months)					0·023	0·008	8·06	0·005	1·024	1·007	1·04
	Sunshine hours: MJJ (2012–2015)					2·189	0·643	11·59	0·001	8·92	2·531	31·45
Farm *F. hepatica*		0·57	0·87	66·8	1st							
	Constant					−6·638	2·329	8·13	0	0	–	–
	Light soils					−1·672	0·779	4·61	0·032	0·188	0·041	0·865
	Soil CU					0·286	0·099	8·43	0·004	1·331	1·097	1·615
	Access to natural water					2·005	0·884	5·15	0·023	7·426	1·313	41·987
	Percentage of fields with bog habitats					1·509	0·548	7·60	0·006	4·524	1·547	13·233

AIC, Akaike information criterion; AUC, area under the curve; CI, confidence interval; HL, Hosmer–Lemeshow; NMR, Null Model Rank; s.e., standard error; MJJ; May –July.

## DISCUSSION

This study is the first to record the on-farm prevalence of *C. daubneyi* in any area within the UK. This initial estimate of *C. daubneyi* prevalence (61%) indicates that it is established in Wales, a finding which is supported by the increasing prevalence of rumen fluke observed by passive veterinary surveillance across the UK since 2010 (VIDA, 2016*a*). The logistic regression models created are also the first models explaining the prevalence of *C. daubneyi* in an area of the UK. Each final model created included both climate and environmental/management factors, and were superior to climate only models at the model selection stage. This indicates the importance of using environmental and management factors alongside climate variables when modelling *C. daubneyi* prevalence at farm level, as is the case with *F. hepatica* (Bennema *et al.* 2011).

Despite our finding that *C. daubneyi* may be very common in Wales; it is not universally known within the Welsh agricultural community. *Calicophoron daubneyi* prevalence was shown to be lower than *F. hepatica* despite the fact that most farms in the study treated against *F. hepatica* and not *C. daubneyi. Calicophoron daubneyi* may therefore be in the process of spreading and colonizing farms and regions in Wales. The high proportion of farms harbouring co-infection of *C. daubneyi* and *F. hepatica* within their livestock (46%) suggests the parasites share a similar geographic range in Wales. Similarities were also observed between *C. daubneyi* and *F. hepatica* risk factors, with environmental features linked to *G. truncatula* presence such as presence/density of boggy habitats, soil factors and water sources important variables in models for both parasites. In contrast to the *C .daubneyi* models, climate variables were absent from the *F. hepatica* model, which may be explained by the importance of environmental factors in previous *F. hepatica* predictive models (McCann *et al.* 2010; Bennema *et al.* 2011).

A negative correlation was recorded between the intensity of infection of each parasite in both cattle herds and sheep flocks. The high levels of *C. daubneyi* and low levels of *F. hepatica* seen in numerous cases could be explained by Welsh farmers treating against *F. hepatica* with anthelmintics not active against *C. daubneyi.* Yet treatment regimen alone does not explain the decreasing EPG levels of *C. daubneyi* seen in the presence of increasing *F. hepatica* as none of the small proportion of participating farms in this study that had recently used oxyclozanide in their livestock had higher EPG levels of *F. hepatica*. This raises potential intriguing questions regarding the epidemiology of each parasite. Despite their apparent preference for the same intermediate snail host, it is unclear whether each parasite's different epidemiology leads to farms being more suited to one fluke species than the other, or whether potential competition between each parasite at the intermediate snail stage occurs. *C. daubneyi* and *F. hepatica* are known to eliminate each other when dually infecting *G. truncatula* (Rondelaud *et al.* 2007) while the presence of other trematode have been shown to suppress *F. hepatica* levels within snail populations (Gordon and Boray, 1970; Whitelaw and Fawcett, 1982; Goumghar *et al.* 2000). If competition does occur, it could explain the reason for variables regarding treatment against *F. hepatica* being a positive predictor for *C. daubneyi* in final models. By treating against *F. hepatica* regularly, especially during spring (a positive predictor for *C. daubneyi* presence in the cattle model), the number of *F. hepatica* eggs shed onto pasture may be reduced, potentially freeing *G. truncatula* snails to be infected with *C. daubneyi*. However, caution must be maintained when interpreting these results, as treatment regularity may be partly determined by historic *F. hepatica* issues, which in turn may be partly determined by the density of *G. truncatula* habitats. Despite this, an increasing efficacy of treatment for *F. hepatica* in France has been suggested as a reason for their recorded increase in paramphistomosis over the past 25 years (Mage *et al.* 2002; Rieu *et al.* 2007). In the UK, the growing threat of triclabendazole resistance (Gordon *et al.* 2012) is causing major issues with *F. hepatica* treatment efficacy (Sargison *et al.* 2016). Thus, it is hypothesized that the varying efficacy of a farm's treatment regimen against *F. hepatica* could determine, which parasite prevails in a competition to dominate a farm's endogenous *G. truncatula* populations and therefore be capable of infecting livestock at high intensities. Furthermore, in an instance where *C. daubneyi* could suppress *F. hepatica* through competition, farms could in theory benefit from the presence of *C. daubneyi*. For example, it is currently believed that a heavy burden of juvenile *C. daubneyi* is required to cause clinical disease in livestock (Mason *et al.* 2012; Millar *et al.* 2012), with adult paramphistomes well tolerated (Zintl *et al.* 2014). In comparison, heavy infections of both juvenile and adult *F. hepatica* are known to cause severe losses in livestock (Dargie, 1987), with production losses also associated with lighter burdens (Schweizer *et al.* 2005). However, further research would be required on both the dynamics of this potential competition, as well as the pathogenicity of both juvenile and adult *C. daubneyi* prior to any implementation of strategies to take advantage of this potential phenomenon.

Differences in the prevalence of *C. daubneyi* between cattle herds and sheep flocks were also observed, with prevalence higher in cattle compared with sheep. This coincides with UK passive veterinary surveillance data (VIDA, 2016*a*) and data from Irish abattoirs and veterinary surveillance (Toolan *et al.* 2015). One suggested reason for the latter finding in Ireland was that sheep graze rougher pasture compared with cattle; however, this seems to be an unlikely factor in our results as there was no significant difference between *F. hepatica* prevalence in cattle and sheep, which indicates a similar exposure to *G. truncatula* habitats for each ruminant species. It has been shown that another Paramphistomum species, *C. microbothrium,* is better suited to infecting cattle compared with sheep, with the parasite having an increased lifespan, and is known to migrate quicker and exhibit increased growth and egg laying ability when infecting cattle (Horak, 1967). *Calicophoron daubneyi* may similarly be better suited to infecting cattle compared with sheep, however, there was no significant difference in the EPG levels seen in positive herds and flocks, in fact the highest EPG levels for *C. daubneyi* in this study was seen in a sheep flock. Ovine paramphistomosis has also been reported in the UK (Mason *et al.* 2012), which indicates that *C. daubneyi* should not be disregarded as a pathogenic parasite of sheep. The importance of cattle in the epidemiology of *C. daubneyi* was further emphasised in each model, where either the total number of cattle or number of heifers or steers present on a farm were selected as positive predictors for *C. daubneyi*. An increasing herd size may increase the sources of *C. daubneyi* eggs shedding onto pasture and thus parasite spread within a farm, but may also lead to an increased risk of buying in the parasite with increasing herd size a potential proxy for herd turnover rate (Reilly and Courtenay, 2007). This was highlighted in the questionnaire data where farms buying in cattle had significantly larger herd sizes. This also highlights the role of biosecurity in disease prevention; with *C. daubneyi* seemingly well-established less than a decade since significant reports of its occurrence in the UK began.

Other variables which could have biosecurity implications that were included as positive predictors in the best performing models were the ‘presence of livestock accessible streams or drainage ditches’ and ‘water flowing from other farms’. The inclusion of these variables may be a measure of the presence of intermediate host snail habitats, with muddy areas surrounding streams and ditches a common habitat for *G. truncatula* similarly to boggy pastures, which was also a positive predictor for *C. daubneyi* in two of the final models. However, streams are also potential movement corridors for *G. truncatula* snails, with data from France suggesting *G. truncatula* snails can travel upstream and contaminate pasture free from livestock with *F. hepatica* metacercariae (Rondelaud *et al.* 2005). The role of streams in the spread of *C. daubneyi* and potentially TCBZ resistant strains of *F. hepatica* in the UK is therefore worthy of further investigation.

The only direct climate variable to be included in all of the final *C. daubneyi* models was sunshine hours. The exact reason for this inclusion is unclear; however, *C. daubneyi* eggs are known to be more dependent on light as a hatching stimulus compared with *F. hepatica* eggs (Chryssafidis *et al.* 2015). If this is the reason for sunshine's inclusion in each model it may suggest that eggs in areas receiving longer levels of sunshine may have higher hatching success rates, leading to increasing lifecycle opportunities. A calculated Ollerenshaw index value for the summer of 2015 was also a positive predictor of *C. daubneyi* in the cattle model. Although its effect on the model was small, potentially due to the limited impact of between-year weather variation on between-farm fluke prevalence (McCann *et al.* 2010), its inclusion may unsurprisingly suggests that climate predictors of fasciolosis may also have predictive power regarding *C. daubneyi.* This may partly explain the higher prevalence of *C. daubneyi* seen in participating farms from western regions of Wales, with western Wales shown to be one area of the UK at most risk of fasciolosis both historically and in future forecasts when using the Ollerenshaw model (Fox *et al.* 2011). A westerly trend of increased rumen fluke detection in passive veterinary surveillance has similarly been observed in Wales and throughout the UK (VIDA, 2016*a*), which would also suggest that the traditional climate drivers of *F. hepatica* prevalence are also important factors for *C. daubneyi.* To enhance our understanding of the relationship between climatic variables and *C. daubneyi* prevalence, it would be necessary to analyse a dataset on a wider scale.

It remains unclear how and why *C. daubneyi* has apparently become so prevalent in the UK in recent years, and questions remain regarding its origins. It is possible that low levels of *C. daubneyi* in the UK may have been overlooked previously due to the perceived presence of *P. cervi* as the dominant Paramphistomatidae sp. in UK livestock (Gordon *et al.* 2013), and its non-pathogenicity at low levels (Zintl *et al.* 2014). In this instance, it could be suggested that a decrease in the popularity of ‘old’ anthelmintic products, including oxyclozanide, following the licencing of TCBZ in the mid 80s would have allowed *C. daubneyi* to ultimately increase its prevalence in UK livestock. There is also a possibility that *C. daubneyi* parasites may have recently been imported into the UK in infected animals, with cattle imports to the UK increasing substantially in the aftermath of the 2001 foot and mouth disease outbreak (Mitchell *et al.* 2005), which coincides with a period where *C. daubneyi* levels in France had just increased significantly (Mage *et al.* 2002). In either or both instances, it is likely that climate change will have played a role in the recent establishment of *C. daubneyi* in the UK. Since 1970, increasing rainfall and temperatures have seen the UK climate become more suitable for *G. truncatula* populations (Fox *et al.* 2011), a factor which has seen increasing reports of fasciolosis (Pritchard *et al.* 2005) and may have had a similar positive effect on *C. daubneyi*. Increasing annual hours of sunshine have also been observed in the UK over the past 50 years (Met-Office, 2016), which may have also contributed to *C. daubneyi* establishment if indeed sunshine duration is a driver for its prevalence. Climate change could also lead to future increases in *C. daubneyi* prevalence due to further climate change forecast of increases in climate suitability for *G. truncatula* (Fox *et al.* 2011).

### Concluding remarks

This study was the first farm level survey for *C. daubneyi* in any area of the UK, with the results indicating *C. daubneyi* is endemic in Wales. The study has also raised important questions regarding differing prevalence levels between ruminant species, and potential competition between *C. daubneyi* and *F. hepatica.* To answer these questions further research on the epidemiology of *C. daubneyi* at a finer and broader scale will be required. Finally, this study produced the first model for *C. daubneyi* in an area of the UK. The predictors identified may be used in future as a basis to further study *C. daubneyi* epidemiology and to develop models of further value to farmers and veterinarians in predicting and combating *C. daubneyi* occurrence and paramphistomosis risk.

## Supplementary material

For supplementary material accompanying this paper visit http://dx.doi.org/10.1017/S0031182016001797. click here to view supplementary material
