# Supplementary material for: Rumen fluke (Calicophoron daubneyi) on Welsh farms: prevalence, risk factors and observations on co-infection with Fasciola hepatica
Source: Parasitology. 2016 Nov 1;144(2):237–47. doi: 10.1017/S0031182016001797 (PMC5300002; doi:10.1017/S0031182016001797)
Supplement: Supplementary file 1 [file S0031182016001797sup001.pdf]

Supplementary table 1: Variables accepted and rejected as candidate variables for inclusion in logistic regression models after univariate analysis

Variable	Source	Significance		
		Farm	Cattle	Sheep
Liver fluke +/-	FEC	*	**	NS
Mt Summer 2015	Ollerenshaw index	NS	*	NS
Mt Summer 2011-15	Ollerenshaw index	NS	ƚ	NS
Mt Winter 2014/15	Ollerenshaw index	NS	NS	NS
Mt Summer 2010/11-2014/15	Ollerenshaw index	NS	NS	NS
Rainfall (April - September) 2015	Met-Office	NS	NS	NS
Rainfall (April - September) 2011-2015	Met-Office	NS	NS	NS
Rain days 1mm (April - September) 2015	Met-Office	NS	NS	NS
Rain days 1mm (April - September) 2011-2015	Met-Office	NS	NS	NS
Mean temperature (April - September) 2015	Met-Office	ƚ	**	NS
Mean temperature (April - September) 2011-2015	Met-Office	ƚ	**	NS
Mean winter min Temperature (3 coldest month) 2014/15	Met-Office	*	**	ƚ
Mean winter min Temperature (3 coldest month) 2011-2015	Met-Office	*	**	ƚ
Growing degree days 2015	Met-Office	NS	**	NS
Growing degree days 2011/15	Met-Office	NS	**	NS
Sunshine May-July 2015	Met-Office	**	**	***
Sunshine May-July 2011/15	Met-Office	**	**	***
Sunshine April-September 2015	Met-Office	**	**	***
Sunshine April-September 2011/15	Met-Office	**	**	***
Soil pH	CEH	*	NS	NS
Soil type (Light)	CEH	NS	NS	NS
Soil type (Heavy)	CEH	*	NS	NS
Soil moisture	CEH	*	NS	ƚ
Soil Cu	BGS	ƚ	ƚ	*
Soil Fe	BGS	NS	NS	NS
Soil P	BGS	NS	*	NS
Soil Pb	BGS	*	NS	NS
Soil Zn	BGS	**	ƚ	ƚ
Dairy cattle (Yes/no)	Questionnaire	NS	NS	NS
Suckler cattle (Yes/no)	Questionnaire	NS	NS	NS
Heifers/Steer cattle (Yes/no)	Questionnaire	NS	NS	NS
Sheep (Yes/no)	Questionnaire	NS	NS	NS
Dairy cattle numbers	Questionnaire	NS	NS	NS
Suckler cattle numbers	Questionnaire	NS	NS	NS
Heifers/Steers cattle numbers	Questionnaire	*	ƚ	ƚ

Sheep numbers	Questionnaire	NS	NS	NS
Total cattle numbers	Questionnaire	*	ƚ	NS
Total LSU	Questionnaire	NS	NS	NS
Purchase replacement heifers/cattle	Questionnaire	NS	NS	NS
Purchase replacement ewes	Questionnaire	NS	NS	NS
Quarantine treatment against *F. hepatica*	Questionnaire	NS	NS	NS
Quarantine treatment against *C. daubneyi*	Questionnaire	NS	NS	NS
Recently Imported livestock	Questionnaire	NS	NS	NS
Purchase store cattle	Questionnaire	**	*	*
Purchase store sheep	Questionnaire	NS	NS	NS
Animals from other farm grazing holding	Questionnaire	NS	NS	*
Animals grazing other farm	Questionnaire	NS	NS	ƚ
Graze common land	Questionnaire	NS	NS	NS
No contact with animals from other farm	Questionnaire	NS	NS	NS
Organic	Questionnaire	NS	NS	NS
Previously organic	Questionnaire	NS	NS	NS
Deer present	Questionnaire	NS	NS	NS
Hares present	Questionnaire	NS	NS	NS
Rabbits present	Questionnaire	NS	NS	NS
Number of yearly treatment against *F. hepatica*	Questionnaire	**	**	NS
Autumn treatment against *F. hepatica*	Questionnaire	NS	NS	NS
Winter treatment against *F. hepatica*	Questionnaire	NS	NS	NS
Spring treatment against *F. hepatica*	Questionnaire	NS	ƚ	NS
Summer treatment against *F. hepatica*	Questionnaire	NS	NS	NS
Use of Oxyclozanide	Questionnaire	NS	NS	NS
Resistance to *F. hepatica* proven	Questionnaire	NS	NS	NS
Resistance to *F. hepatica* suspected	Questionnaire	NS	NS	NS
Farm area (ha)	Questionnaire	NS	NS	NS
Stocking density LSU/ha	Questionnaire	NS	NS	NS
Soil moisture	Questionnaire	NS	NS	NS
Flooding occurrence	Questionnaire	NS	NS	NS
Presence of bog habitats	Questionnaire	*	*	NS
Presence of wet habitats	Questionnaire	NS	NS	NS
Presence of stream/drainage ditch habitats	Questionnaire	*	NS	*
Presence of pond habitats	Questionnaire	NS	NS	NS
Percentage of fields containing bog habitats	Questionnaire	NS	NS	NS
Percentage of fields containing wet habitats	Questionnaire	NS	NS	NS
Percentage of fields containing stream/drainage ditch habitats	Questionnaire	NS	NS	NS
Percentage of fields containing pond habitats	Questionnaire	NS	NS	NS
Grazing season length (cattle only)	Questionnaire	N/A	ƚ	N/A
Natural water access for livestock	Questionnaire	NS	NS	NS
Artificial water access for livestock	Questionnaire	NS	NS	NS
Water flowing from other farm	Questionnaire	NS	ƚ	NS

ƚ P<0.10, * P<0.05, ** P<0.01, *** P<0.001
